# Bioactive Self-Nanoemulsifying Drug Delivery Systems (Bio-SNEDDS) for Combined Oral Delivery of Curcumin and Piperine

**DOI:** 10.3390/molecules25071703

**Published:** 2020-04-08

**Authors:** Mohsin Kazi, Ahmad A. Shahba, Saad Alrashoud, Majed Alwadei, Abdelrahman Y. Sherif, Fars K. Alanazi

**Affiliations:** 1 Department of Pharmaceutics, College of Pharmacy, King Saud University, Riyadh-11451, Saudi Arabia; saadr@ksu.edu.sa (S.A.); ph.alwadei@gmail.com (M.A.); abdelrhmanshreef@gmail.com (A.Y.S.); falanazi1@ksu.edu.sa (F.K.A.); 2 Kayyali Chair for Pharmaceutical Industries, Department of Pharmaceutics, College of Pharmacy, King Saud University, Riyadh 11451, Saudi Arabia; shahba@ksu.edu.sa

**Keywords:** curcumin and piperine, combination therapy, solidification technique, self-nanoemulsifying drug delivery systems (SNEDDS), dissolution improvement, bio-SNEDDS

## Abstract

**Background**: Bioactive oils of natural origin have gained huge interests from health care professionals and patients. **Objective**: To design a bioactive self-nanoemulsifying drug delivery system (Bio-SNEDDS) comprising curcumin (CUR) and piperine (PP) by incorporating bioactive natural oils in the formulation. **Methods**: The self-emulsifying properties of apricot, avocado, black seed and *Zanthoxylum rhetsa* seed oils were screened within various SNEDDS formulations. Each liquid SNEDDS formulation was loaded with both CUR and PP. The optimal liquid SNEDDS were solidified using Aeroperl^®^ and Neusilin^®^ at 1:1 *w*/*w* ratio. Liquid and solid SNEDDS were characterized by droplet size analysis, equilibrium solubility, scanning electron microscopy, X-ray powder diffraction, differential scanning calorimetry, and Fourier transform infrared spectroscopy. In-vitro dissolution studies were performed to evaluate the efficiency of CUR and PP release from solid Bio-SNEDDS. **Results**: The liquid SNEDDS comprised of black seed oil exhibited excellent self-emulsification performance, low droplet size along with transparent appearance. The inclusion of the cosolvent Transcutol P improved the solubilization capacity of both CUR and PP. The liquid SNEDDS were efficiently solidified using the two adsorbents and presented the drugs within amorphous state. In particular, SNEDDS comprised of black seed oil/Imwitor988/Transcutol P/Cremophor RH40 (20/20/10/50) and when solidified with Neusilin showed enhanced CUR and PP release (up to 60% and 77%, respectively). In addition, this formulation efficiently delivers the highly bioactive black seed oil to the patient. Conclusions: The optimized Bio-SNEDDS comprising black seed oil showed outstanding self-emulsification characteristics along with enhanced CUR/PP dissolution upon solidification.

## 1. Introduction

The demand for natural formulation active/inactive ingredients is increasing due to the increased risk of side effects posed by the synthetic compounds. Pharmaceutical ingredients of plant origin are generally safer as they produce less toxic metabolites. Various diseases (such as diabetes, cancer, stroke, Alzheimer’s and atherosclerosis), have been safely treated with natural entities and antioxidant-based formulations [1]. Nowadays, bioactive natural oils play vital roles in the development of new drugs, especially for antitumor, antimicrobial, and psychoactive agents. The outstanding pharmacological benefits of these ingredients attract scientists to investigate more on the characterization of their biological profile. Such interesting ingredients increase the possibility of obtaining new targeted therapies for many challenging diseases [2].

**Figure 1:**
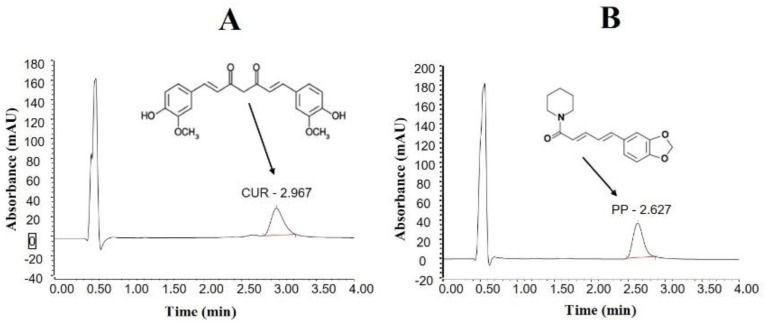
UHPLC chromatograms and chemical structures of (**A**) curcumin and (**B**) piperine in a self-nanoemulsifying lipid formulation sample. CUR denotes curcumin and PP: piperine.

Curcumin (CUR, Figure 1A), a highly lipophilic bioactive constituent extracted from the rhizomes of the herb *Curcuma longa L*., has been reported to exhibit various biological and pharmacological effects (Table 1). In animal models, oral administration of CUR inhibited cancer of lung [3], skin [4], neck and head [5], oral [6], hepatocellular carcinoma [7]**,** lymphomas, mammary tumors and leukemias [8]. Because of its attractive properties, CUR is marketed in several countries worldwide and in different dosage forms. However, despite its promising pharmacological effects and safety, the clinical use of CUR has been limited by its poor bioavailability, which was correlated to its low aqueous solubility, extensive hepatic and intestinal metabolism, and fast systemic elimination [9]. Even at a high dose, serum concentration of CUR was very low (only 1% in rat) [10]. 

These limitations should find a solution through using novel drug delivery systems. Therefore, it is very important to increase CUR aqueous solubility and decrease its metabolic clearance simultaneously. An interesting approach for improving the delivery of CUR is co-administration with piperine (PP, Figure 1B). PP is a major component of black pepper that exhibits several beneficial biological effects. PP acts as hepatic and intestinal glucuronidation inhibitor and has been reported to enhance the extent of absorption, serum concentration, and bioavailability of CUR in rats as well as humans [11]. In particular, concomitant administration of PP along with CUR produced a 2000% increase in CUR bioavailability compared to CUR alone in humans. In addition, the combination of PP with CUR showed significant potentiation of its neurotransmitter enhancing (serotonin and dopamine), anti-immobility, and monoamine oxidase inhibitory effects as compared to the CUR effect alone [12]. These studies provide a scientific rationale for the co-administration of PP with CUR to enhance the latter bioavailability and therapeutic efficacy.

Self-emulsifying, microemulsifying and nanoemulsifying drug delivery systems (SEDDS/SMEDDS/SNEDDS) are highly effective in enhancing the aqueous solubility, dissolution and bioavailability of poorly-water soluble drugs [13,14]. These lipid-based systems are composed of isotropic mixtures of oils, surfactant, cosurfactants and/or cosolvents. According to the “Lipid Formulation Classification System”, the oil proportion might range from 100% (Type I), 40–80% (Type II and IIIA), <20% (Type IIIB) or even 0% (Type IV) [15,16]. The utilized oil in these systems can be natural, synthetic or semi-synthetic. In the conventional SEDDS, oils are used as inactive ingredients (lipophilic solubilizers) to increase the loading of poorly-water soluble drugs. Several articles have shown the beneficial role of conventional SEDDS enhancing the aqueous solubility, absorption and bioavailability of CUR or PP single administration [10,17,18,19]. Furthermore, the combined CUR-PP SMEDDS improved CUR water solubility, stability and anti-colitis activity [18]. However, none of these articles explored the potential of incorporating bioactive oils into CUR-PP SNEDDS formulation. Due to the outstanding health benefits of bioactive natural oils, it is worthy to investigate the feasibility of incorporating them in SNEDDS formulation. This strategy could lead to development of a new generation of novel bioactive lipid-based formulations called Bio-SNEDDS, which offer dual benefits; enhancing the dissolution and bioavailability of poorly-water soluble drugs along with delivering such beneficial bioactive oils to the patient. 

In the current study, four bioactive oils namely, black seed oil (BSO), avocado oil (AVO), apricot oil (APO) and *Zanthoxylum rhetsa* seed oil (ZRO) were investigated in the formulation of Bio-SNEDDS for CUR and PP delivery. In addition, these bioactive oils have several valuable nutritive and therapeutic effects on human health (Table 1). Furthermore, to avoid lipid oxidation and improve the overall formulation stability, the optimized liquid SNEDDS were solidified using the two adsorbent grades Neusilin^®^ US2 and Aeroperl^®^ 300.

The proposed formula provides a novel strategy to develop Bio-SNEDDS formulations of CUR–PP using bioactive oil excipients. The co-delivery systems were characterized in terms of equilibrium solubility, appearance, droplet size, zeta potential and in-vitro dissolution.

## 2. Results

### 2.1. UHPLC Analysis for CUR and PP

The developed UHPLC method showed good selectivity for simultaneous quantification of CUR and PP in lipid-based systems. CUR and PP were eluted at 2.967 and 2.627 min at wavelengths 428 and 338 nm, respectively (Figure 1). The developed method showed good linearity for CUR and PP (r^2^ = 0.997 and 0.999, respectively) over the concentration range of 25 and 2500 ng/ml.

### 2.2. Characterization of Liquid CUR-PP SNEDDS

#### 2.2.1. Equilibrium Solubility of CUR and PP in SEDDS/SNEDDS Formulations

The designed lipid-based formulations showed considerable variability in CUR and PP solubilities in response to modification of excipients type and ratio (Table 2). The equilibrium solubility of CUR and PP in the anhydrous formulations ranged from 19 to 38 and 37 to 48 mg/g, respectively. F6 (Type IIIB LFCS formulation) showed the highest CUR solubility while F5 (Type II LFCS formulation) showed the highest PP solubility.

#### 2.2.2. Appearance and Homogeneity

**Figure 2:**
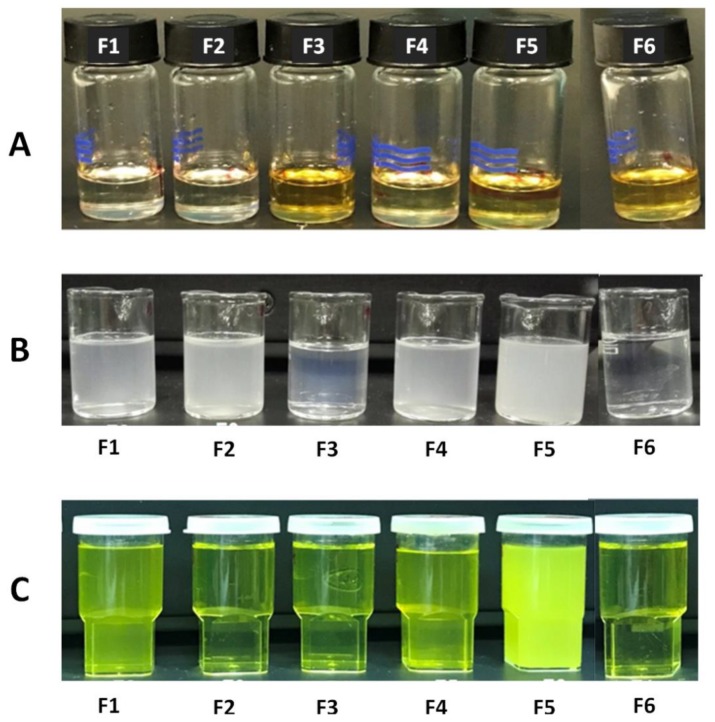
Appearance of (**A**) anhydrous SNEDDS after loading with CUR and PP, (**B**) drug-free SNEDDS after 1:100 aqueous dilution and (**C**) CUR-PP loaded SNEDDS after 1:1000 aqueous dilution.

The designed anhydrous SNEDDS formulation showed good mutual miscibility and homogeneity even after CUR and PP loading (Figure 2A). Upon aqueous dispersion, all the drug-free SNEDDS formulations showed excellent homogeneity and spontaneity, particularly F3 and F6, which showed transparent appearance (Table 3 and Figure 2B). The aqueous dispersion of drug-loaded SNEDDS revealed the yellowish characteristic tint of CUR. Most importantly, F2, F3 and F6 showed transparent appearance (Figure 2C). On the other hand, F5 showed turbid appearance upon aqueous dispersion of both drug-free and drug-loaded formulations.

#### 2.2.3. Droplet Size and Zeta Potential

**Figure 3:**
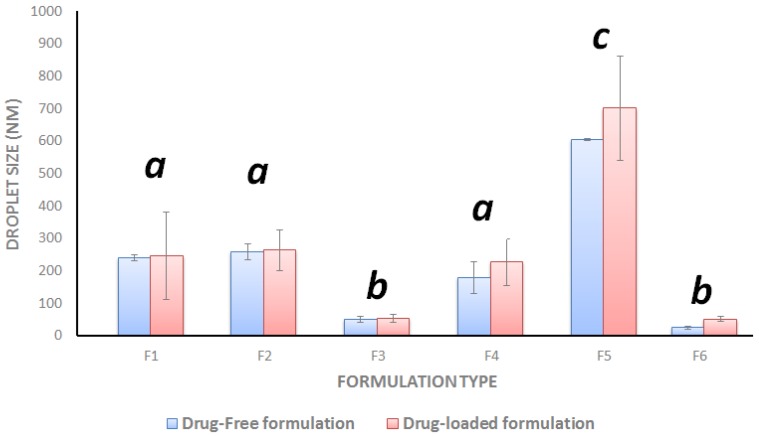
Influence of the drug loading on formulation droplet size. Different superscripted letters indicate statistical significance (*p* < 0.05) between formulations.

The droplet size of drug-free SNEDDS ranged from (25–604 nm) where the F6 (Type IIIB system) showed the lowest droplet size and F5 (Type II system) showed the largest droplet size (Figure 3). Similarly, the droplet size of drug loaded SNEDDS containing CUR and PP ranged from 51–701 nm. Overall, there was no significant increment in droplet size upon CUR and PP loading (Figure 3).

**Figure 4:**
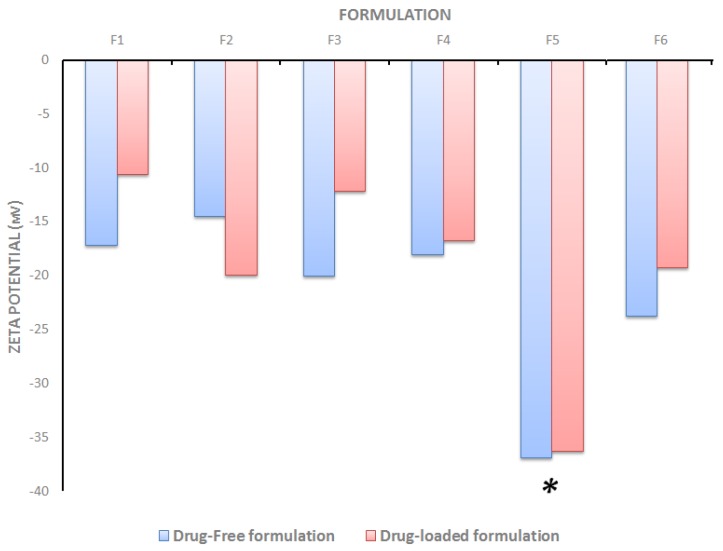
Influence of the drug loading on formulation zeta potential. * denotes statistical significance (*p* < 0.05) from all other formulations.

On the other hand, the zeta potential of drug-free SNEDDS ranged from (–14.5 to –36.9 mV) indicating a good physical stability of the formed emulsion particularly with F5 (Figure 4). On the other hand, the zeta potential value of CUR-PP loaded SNEDDS ranged from (–10.6 to –36.4 mV). Overall, there was no significant change in zeta potential value upon CUR-PP loading (Figure 4). 

#### 2.2.4. Dynamic Dispersion Studies

**Figure 5:**
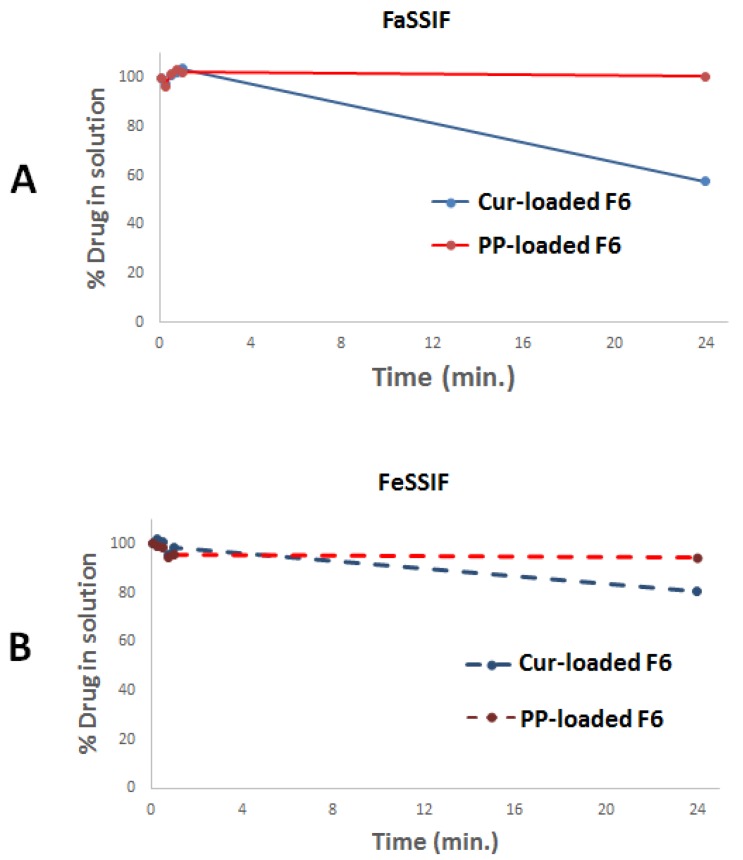
The percent of CUR and PP remaining in solution during 24 h time after dilution in (**A**) fasted and (**B**) fed state simulated intestinal fluid (FaSSIF and FeSSIF, respectively). SNEDDS was represented by F6 (BSO/988/TcP/CrRH40 [20/20/10/50]), CUR: curcumin and PP: piperine.

In dynamic dispersion studies the emphasis should be on detecting undesirable drug precipitation rather than dissolution. Upon aqueous dispersion in FaSSIF, SNEDDS (represented by F6) maintained >57% CUR and ≈100% PP in solution up to 24 hrs. While in the case of FeSSIF, SNEDDS maintained >80% CUR and >94% PP in solution up to 24 hrs (Figure 5). 

### 2.3. Characterization of CUR-PP Solid SNEDDS

#### 2.3.1. Scanning Electron Microscopy

**Figure 6:**
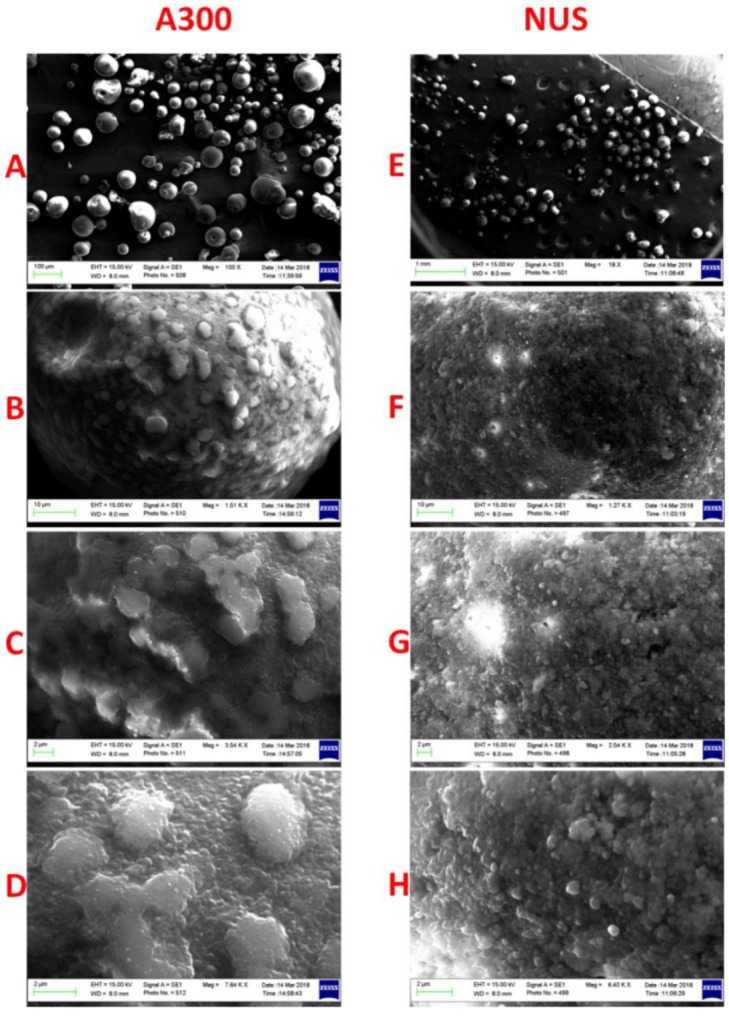
SEM images of (**A**–**D**) A300 representative solidified SNEDDS and (**E**–**H**) NUS representative solidified SNEDDS at ascending magnifications (100–7640× and 18–6400×, respectively).

SEM images showed that the liquid formulations were successfully solidified using A300 and NUS with minimal agglomeration between the solid particles (Figure 6A,E). A300-solidified SNEDDS showed multiple projections on the particle surface which could be attributed to the adsorption of SNEDDS on the outer surface of the adsorbent (Figure 6B–D). On the other hand, NUS-solidified SNEDDS presented less projections and a smoother surface (Figure 6F–H).

#### 2.3.2. Differential Scanning Calorimetry (DSC)

**Figure 7:**
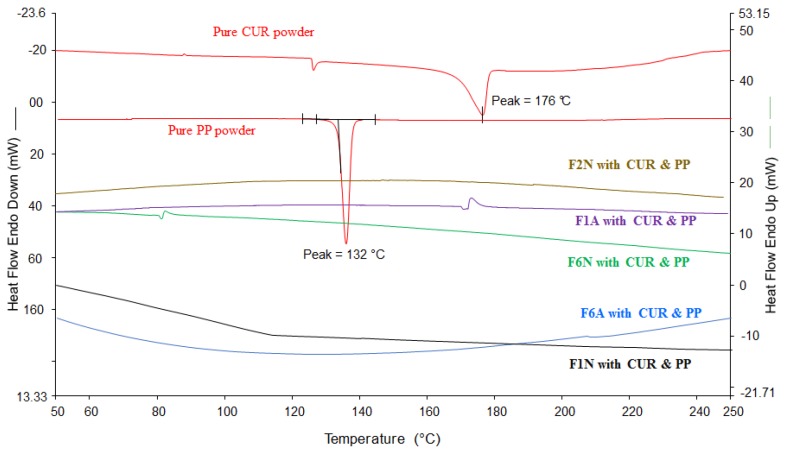
DSC of pure CUR, pure PP and CUR-PP loaded solidified SNEDDS formulations.

Pure PP and CUR exhibited sharp endothermic peaks at 132 and 176 ºC, respectively (Figure 7), which confirm the crystalline state of both drugs. Upon drug loading within the SNEDDS and subsequent solidification with adsorbents, CUR and PP peaks were completely disappeared from all the tested solid SNEDDS. Due to the low drug concentration within solid SNEDDS, it was difficult to decide whether the CUR and PP peaks disappearances were owing to drug transformation into an amorphous state or to significant drug dilution within the formulation [13,40].

#### 2.3.3. X-Ray Powder Diffraction (XRPD)

**Figure 8:**
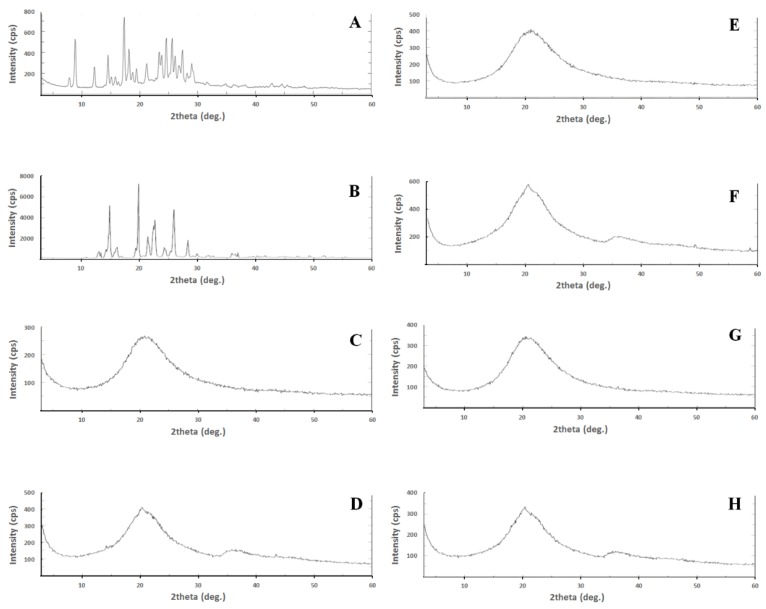
XRD of (**A**) pure CUR, (**B**) pure PP, (**C**) F1A, (**D**) F1N, (**E**) F2A, (**F**) F2N, (**G**) F6A and (**H**) F6N.

The XRPD findings were in good agreement with the DSC results. Pure CUR and PP showed characteristic X-ray diffraction peaks particularly at 3° to 30° (2θ) (Figure 8). In contrast, all the CUR-PP loaded S-SNEDDS showed complete absence of CUR and PP diffraction peaks. Therefore, XRPD data together with DSC data supports that CUR and PP were transformed into an amorphous state within solid SNEDDS [13,40].

#### 2.3.4. Fourier Transform Infrared Spectroscopy (FTIR)

**Figure 9:**
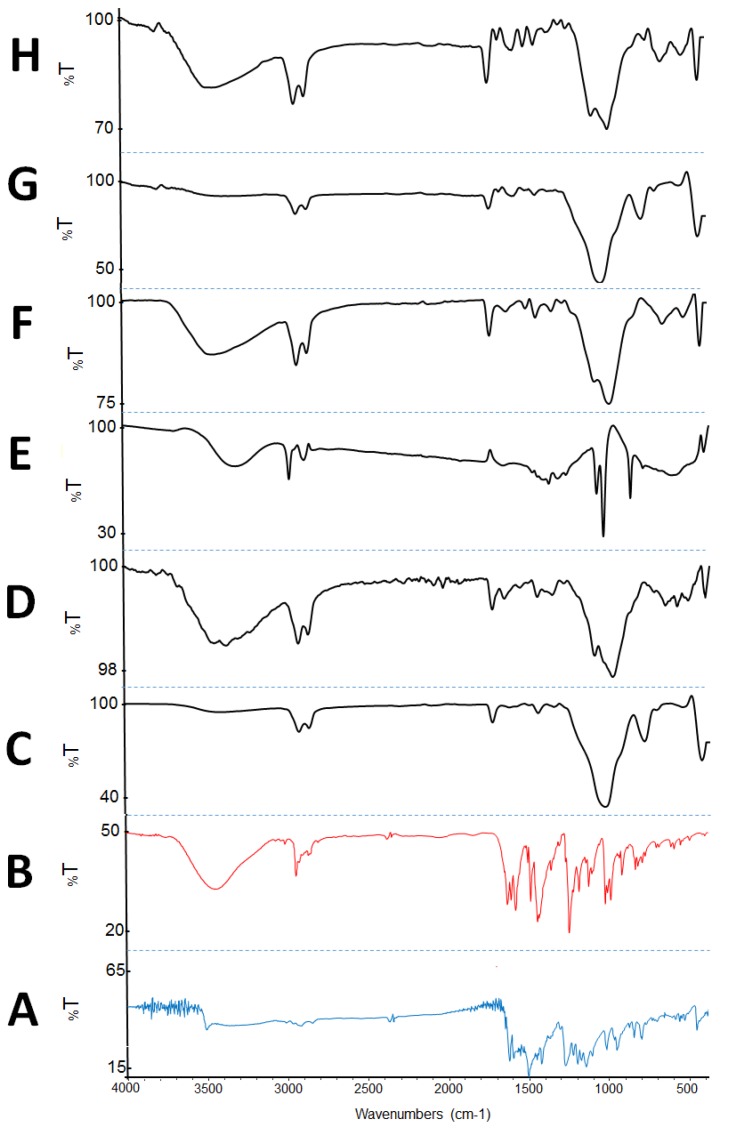
FTIR results of (**A**) pure CUR, (**B**) pure PP, (**C**) F1A, (**D**) F1N, (**E**) F2A, (**F**) F2N, (**G**) F6A and (**H**) F6N.

FTIR was performed to check for any possible drug excipient chemical interaction. The IR spectrum of CUR (Figure 9A) was characterized by principal absorption peaks at 3510, 2345, 1628, 1510, 1429, 1282, 1026, 963, 856, 808 and 458 cm^−1^. While, the IR spectrum of PP (Figure 9B) was characterized by principal absorption peaks at 3448, 2940, 1635, 1611, 1509, 1492, 1365, 1253, 1194, 1134, 1018, 997, 929, 847 and 831 cm^−1^. The FTIR spectra of various solid SNEDDS (Figure 9C–H) suggest that, generally, there was no shift of the drug peaks in the tested solid SNEDDS formulations [41]. It was proposed that CUR and PP peaks were masked by the formulation/carrier absorption peaks due to the very low (<2%) drug concentration within the solid SNEDDS. 

### 2.4. In-Vitro Dissolution Study

#### 2.4.1. Influence of SNEDDS

**Figure 10:**
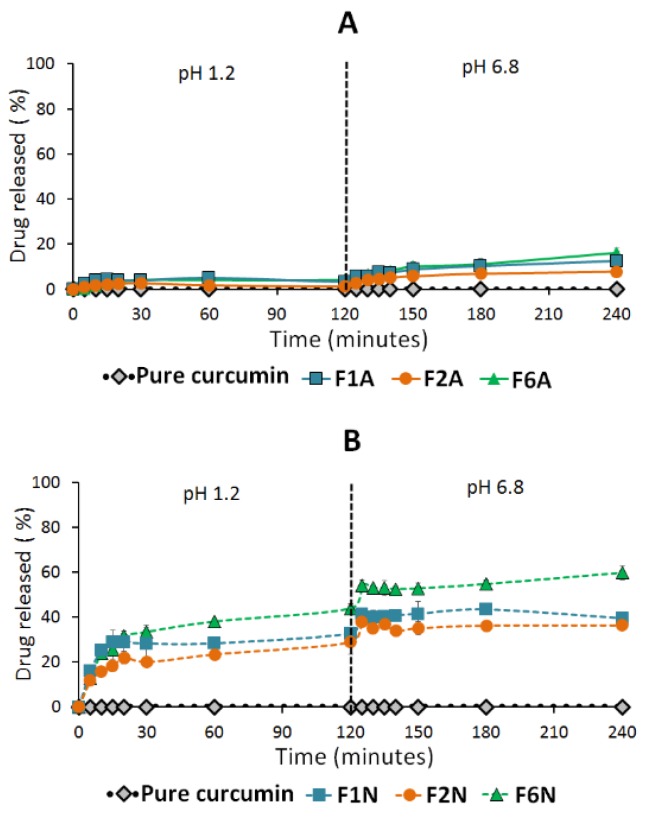
Influence of formulation type and pH on curcumin release from solid SNEDDS. (**A**) Denotes A-300 and (**B**) NUS solidified SNEDDS. The dashed line indicates the time point at which the dissolution media was shifted from pH 1.2 to pH 6.8. Data are expressed as mean ± SD. Details of the formulation compositions are presented in Table 6.

**Figure 11:**
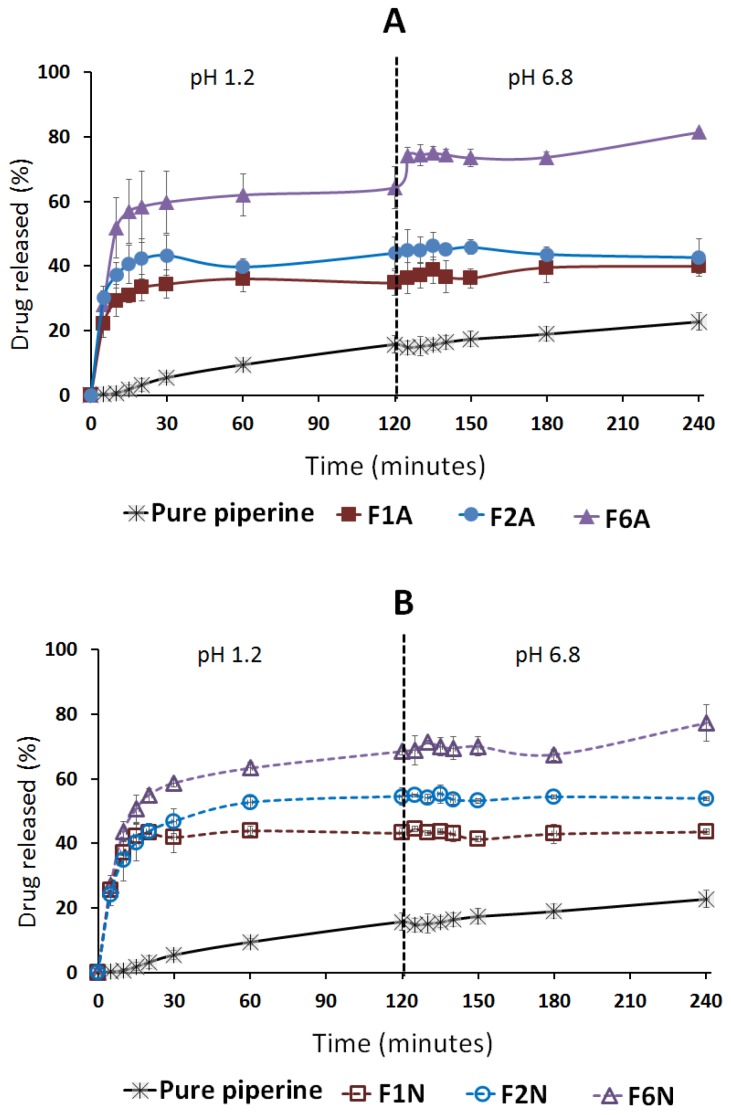
Influence of formulation type and pH on piperine release from solid SNEDDS. (**A**) Denotes A-300 and (**B**) NUS solidified SNEDDS. The dashed line indicates the time point at which the dissolution media was shifted from pH 1.2 to pH 6.8. Data are expressed as mean ± SD. Details of the formulation compositions are presented in Table 6.

Pure CUR showed negligible (≈0%) drug dissolution at both SGF and SIF (Figure 10) while a maximum of 23% drug dissolution was achieved in the case of pure PP powder (Figure 11). On the other hand, all the solid SNEDDS showed significant (*p* < 0.05) enhancement of CUR and PP release at both dissolution media (Figure 10 and Figure 11). In particular, F6N showed significantly (*p* < 0.05) higher CUR release compared to F1N and F2N (Table 4). Similar results were presented for PP release where F6A/F6N showed significantly (*p* < 0.05) higher PP release compared to F1A/F1N and F2A/F2N, respectively (Figure 11).

#### 2.4.2. Influence of pH

On the other hand, the dissolution data revealed that A300-based SNEDDS (F1A, F2A and F6A) showed 1–4% CUR release in SGF (pH 1.2), which was significantly (*p* < 0.05) increased to 8–16% release upon shifting to SIF (pH 6.8) (Figure 10A). Similarly, NUS-based SNEDDS (F1N, F2N and F6N) showed 29–44% CUR release in SGF, which was significantly (*p* < 0.05) increased to 36–60% CUR release upon shifting to SIF (Figure 10B). Generally, the amount of CUR release was significantly (*p* < 0.05) increased upon shifting to SIF. In contrast, PP release was less affected than CUR by pH change from SGF to SIF. NUS-based SNEDDS showed no significant change in PP release upon shifting from SGF to SIF (Figure 11A–B).

#### 2.4.3. Influence of Adsorbent

**Figure 12:**
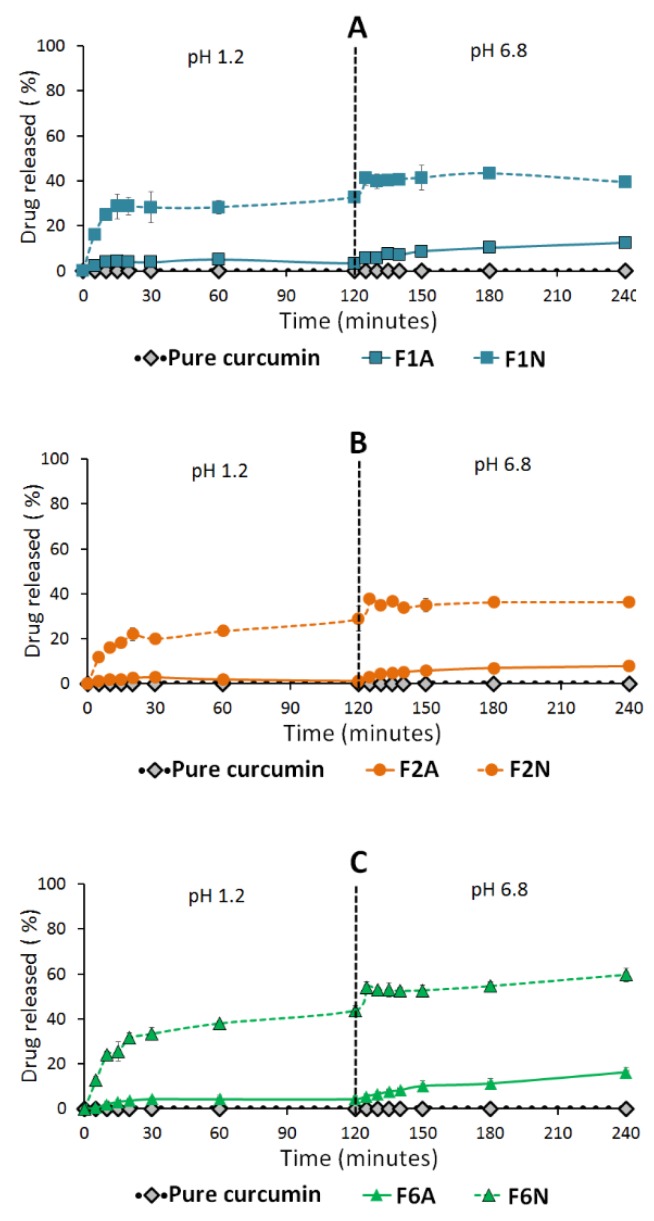
Influence of adsorbent type on curcumin release from solid SNEDDS. (**A**) Denotes F1, (**B**) F2 and (**C**) F6 solidified SNEDDS. The dashed line indicates the time point at which the dissolution media was shifted from pH 1.2 to pH 6.8. Data are expressed as mean ± SD. Details of the formulation compositions are presented in Table 6.

**Figure 13:**
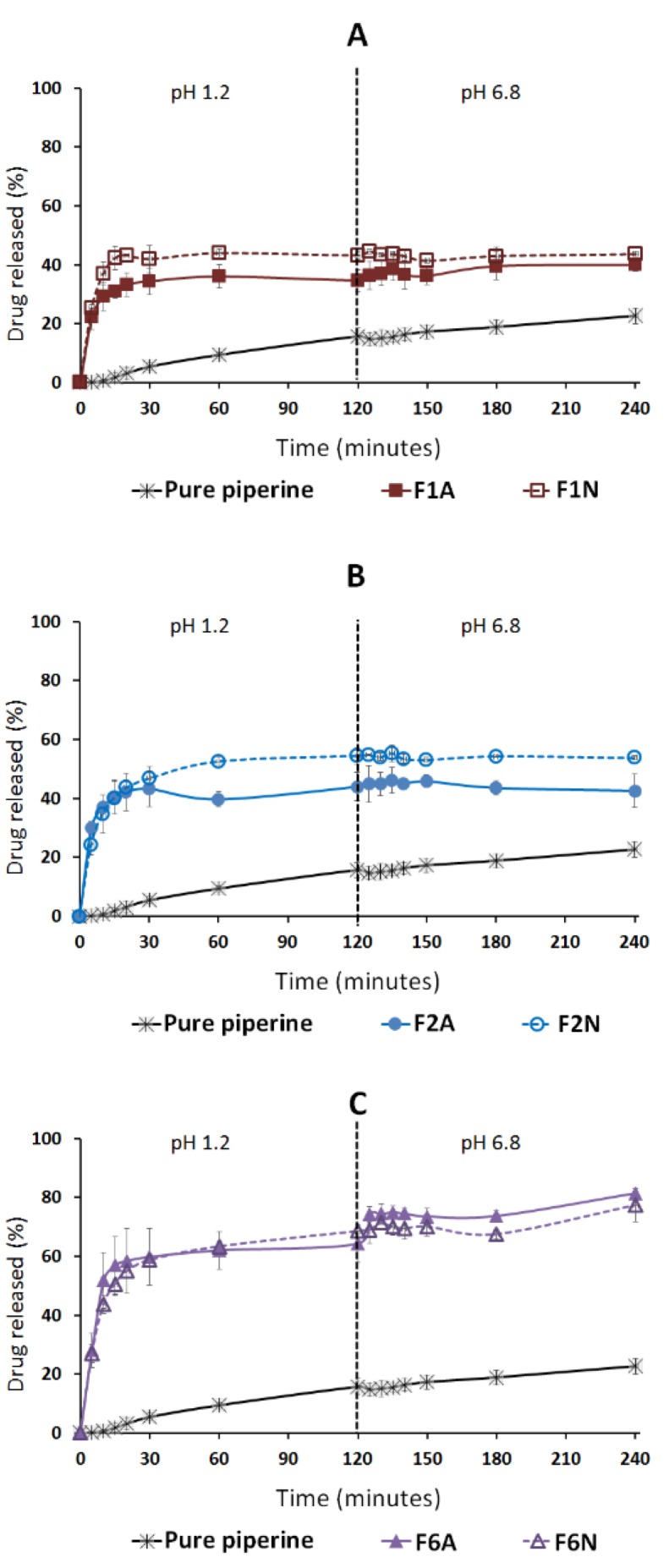
Influence of adsorbent type on piperine release from solid SNEDDS. (**A**) Denotes F1, (**B**) F2 and (**C**) F6 solidified SNEDDS. The dashed line indicates the time point at which the dissolution media was shifted from pH 1.2 to pH 6.8. Data are expressed as mean ± SD. Details of the formulation compositions are presented in Table 6.

All NUS-SNEDDS formulations (F1N, F2N and F6N) showed significantly higher CUR release compared to A300-SNEDDS (F1A, F2A and F6A), respectively (Figure 12). On the other hand, F6N showed no significant difference in PP release compared with F6A (Figure 13). While, F1N and F2N showed significantly higher PP release compared to F1A and F2A, respectively, which is in good agreement with CUR release data (Figure 13 and Table 4). 

## 3. Discussion

L-SNEDDS have demonstrated potential enhancement in the solubility of poorly water-soluble drugs and consequently improved drug bioavailability [15]. However, L-SNEDDS suffer from several stability limitations such as rancidity, incompatibility with capsule shell, risk of formulation leakage, and the possibility of drug precipitation [42,43,44,45]. Furthermore, some drugs might undergo chemical degradation in the presence of SNEDDS components such as oils and related excipients [45]. Solidification of L-SNEDDS possesses high potential to overcome such limitations along with retaining the solubilization benefits of L-SNEDDS. 

Various CUR-PP loaded L-SNEDDS were prepared and the most potential candidates were solidified using the two adsorbents A300 and NUS. In order to obtain an optimum SNEDDS formula, six formulations were screened against their droplet size, zeta potential and equilibrium drug solubility parameters [13]. CUR and PP are poorly-water soluble drugs with partition coefficient values; log*p* ≈ 3 and 2.25, respectively [17,46]. The intermediate log*p* values reveal the expected affinity of the two drugs towards cosolvents and polar oils, which was confirmed by the high CUR and PP solubility in (F6, LFCS Type IIIB systems) (Table 2). However, PP showed high solubility also in Type II and Type IIIA systems that might be correlated to the presence of ZRO oil within these formulations. In particular, CUR implies higher solubility in the co-solvent Transcutol P (TcP), which reveals the fact that CUR has a hydrophobic moiety with less affinity towards Type I and Type II systems that contain significant amount of lipophilic materials [15,47]. 

Droplet size distribution is one of the significant parameters affecting the fate of emulsions in-vivo because it can influence the rate and extent of drug release [48]. The smaller the globule size of the emulsion, the larger the surface area provided for drug absorption. The Z-average size of drug-free and drug-loaded F6-BSO:I988:TcP (2:2;1)/CrRH40 [1:1] was found to be 25 and 51 nm, respectively. The ultra-low droplet size of F6 was highly desirable and was attributed to one or more of the following reasons below:

(a) The high proportion of hydrophilic excipients in the formulation F6 (Type IIIB system) [15].
(b) The inclusion of BSO, which possess good self-emulsification properties as confirmed by the significantly (*p* < 0.05) lower droplet size of both formulations containing BSO (F3 and F6, Figure 3). Similar low droplet size results were reported with other SNEDDS systems containing BSO [46].
(c) The inclusion of the water soluble cosolvent TcP in the formulation.
(d) The inclusion of the highly hydrophilic surfactant Cr-RH40 that has higher HLB (14–16) compared to HCO-40 (12.5) and T85 (11).

Emulsion droplet charge is another parameter in evaluating emulsification efficiency [49]. The significance of zeta potential value could be related to the stability of colloidal dispersions. Colloids with high zeta potential (negative or positive) are electrically stabilized and vice versa. In the current study, F5 showed the highest magnitude of zeta potential values, which gives an indication of system stability and could be attributed to the presence of non-ionic surfactants, adsorption of anionic species (such as hydroxyl ions from the water) to the droplet surfaces, or the existence of some anionic impurities in the surfactant (such as free fatty acids) [50,51] (Figure 4). 

SNEDDS (represented by F6) showed a maximum of 43% CUR precipitation in FaSSIF media, which was reduced to 20% CUR precipitation upon shifting to FeSSIF (Figure 5). These data confirm that FeSSIF was able to minimize CUR precipitation, which is attributed to its higher contents of bile salt/phospholipids, thus playing a vital role in solubilizing hydrophobic and lipophilic molecules [52]. On the other hand, the representative F6-SNEDDS showed less PP precipitation compared to CUR. Overall, a maximum of 6% PP was precipitated in both FaSSIF and FeSSIF media (Figure 5). This could be attributed to the higher affinity of PP towards F6 SNEDDS components (Table 6). 

Solid SNEDDS powders were successfully obtained from liquid SNEDDS formulations by adsorption to solid carriers. The solid SNEDDS characterization revealed the presence of the CUR and PP in a molecularly dissolved amorphous state, within the SNEDDS formulation. These data confirm that both CUR and PP were completely solubilized within S-SNEDDS and that the solidification process did not trigger drug precipitation [13,41]. In addition, there was no obvious sign of chemical interaction between the drugs and the formulations.

The in-vitro dissolution studies revealed that both drugs are hydrophobic, in particular, CUR showed negligible drug dissolution at both SGF and SIF environments. These data emphasize the need for enhancing the oral CUR delivery by proper formulation design. Interestingly, CUR-PP solid SNEDDS showed superior (*p <* 0.05) dissolution enhancement of both drugs at SGF and SIF. This was owing to the efficient self-nanoemulsification process that formed a favorable environment to maintain the drug solubilized, within the nano-sized oil droplets, upon exposure to GI fluids. In particular, F6 showed significantly higher CUR/PP dissolution efficiency compared to F1, F2 and pure drug powder (Figure 10 and Figure 12, and Table 4). The superiority of F6 formulation could be attributed to one or more of the following reasons: 

(1) F6 is LFCS Type IIIB system (enhanced self-emulsification efficiency) compared to F1 and F2, which are Type IIIA systems;

(2) The inclusion of BSO (F6) which possesses excellent self-emulsification properties compared to APO (F1) and AVO (F2) (Figure 2 and Figure 3);

(3) The inclusion of the cosolvent TcP in F6, which significantly increased the CUR and PP solubility within the SNEDDS;

(4) The significantly lower droplet size of F6 compared to F1 and F2.

(5) The inclusion of Cr-RH40 that possesses superior self-emulsification properties as revealed by a previous study that compared the performance of Cr-RH40 and HCO-40 by fixing the oil, cosurfactant and cosolvent and only varying the surfactant type within the formulation. The study revealed that Cr-RH40-based SNEDDS showed excellent (>80%) drug release and lower droplet size compared to HCO-40 counterparts [46]. These findings were correlated with the higher HLB value of Cr-RH40 (HLB = 14–16) compared to HCO-40 (HLB = 12.5).

The increase in CUR release upon shifting to SIF could be owing to the weak acid property of CUR [53]. In contrast, the release of the weak base PP was less affected than CUR by shifting into SIF. On a general basis, A300 presented lower CUR and PP release compared to NUS. This finding could be strongly correlated to the pore size distribution of each adsorbent. A300 is predominately mesoporous (2–50 nm pore size) with the majority of pores ranging from ≈10–30nm (Table 6) [54]. In the current study, the three optimized SNEDDS formulations produce globules with diameters 51–263 nm which are larger than the diameter of the majority of A300 pores. Therefore, it is difficult for such systems to undergo complete emulsification inside the predominant mesoporous region, which hinders complete drug release and solubilization [55]. On the other hand, NUS is predominantly macroporous (>50 nm pore size) with the majority of pores ranging from ≈700–950 nm (Table 6). Moreover, previous SEM images of plain NUS suggested a kind of surface porosity [54]. The significantly larger pores provide the required room to undergo complete emulsification and hence enhanced drug solubilization. It is worth mentioning that the negative influence of A300 was more pronounced (*p <* 0.05) in CUR compared to PP. The significant difference between CUR and PP release from the same adsorbent could be attributed to the strong physical bonds developed between A300 and CUR, which hindered drug release upon exposure to GI fluids. Similar results were observed with the adsorbents Syloid and NUS, and could be correlated with one or more the following factors: smaller pore size, longer pore channels and/or developed hydrogen bonding between the drug and adsorbent [46,54,55,56]. 

In overall assessment, F6N was selected as the optimal CUR-PP SNEDDS formulation due to the high CUR/PP solubility, lower droplet size, good dispersion results (of its liquid form) along with superior CUR-PP release from solid SNEDDS. Most importantly, F6N comprised the bioactive oil BSO. In several Islamic and Arabic countries, black seed is considered as one of the greatest forms of herbal healing medicine. Black seed contains over 100 phytochemical constituents which work together to produce a synergetic effect supporting the immune system and strengthening the body’s constitution. A recent review revealed that black seed (along with its oil) is a multi-disciplinary remedy that can successfully treat over 129 different types of human ailments [33,34]. In particular, black seed oil has a rich composition of several valuable components that play a vital part in forming prostaglandin (PG) E1, which balances and strengthens the immune system against infections, allergies and chronic illnesses [33]. Many therapeutic properties of this plant were suggested to be due to the presence of thymoquinone as a major bioactive component of the essential oil [34]. In addition, black seed oil contains antioxidants that protect the body from free radicals. BSO is also a tremendous source of essential fatty acids [33]. Accordingly, F6N offers a potential oral dosage form for combined oral delivery of CUR-PP along with the bioactive BSO. 

## 4. Materials and Methods

### 4.1. Plant Material

#### 4.1.1. Black Seed Oil (BSO)

##### Seed Collection and Extraction 

The seeds of *Nigella sativa* (*N. sativa*) Linn. (black seeds), family Ranunculaceae were collected from the Southwest part of Bangladesh in the month of March. Seeds (500 gm) were cleaned with fresh water and sundried to remove any moisture. Then the seeds were cold pressed and the oil was filtered and stored in a screw capped amber glass bottle for further use.

##### BSO Standardization

One the principle bioactive constituents of *N. sativa* from its volatile oil is thymoquinone (2-isopropyl-5-methylbenzo-1, 4-quinone) (THQ) with a chemical structure of C10H12O2 and was, therefore, used to standardize BSO. THQ stock solution (100 μg/mL) was used as a reference solution for standardization of BSO. Serial THQ concentrations (0.1–50 μg/mL) were prepared and the actual THQ amount in BSO was calculated based on the THQ calibration curve. Accurately, 1 mL of BSO was separately dissolved in 10 mL solvent within volumetric flasks, filtered and used for analysis. The amount of thymoquinone (THQ) present in the black seed oil was 20%–50%, which matches with the reported standard THQ amount in BSO [46,57].

#### 4.1.2. Zanthoxylum Rhetsa Seed Oil (ZRO)

##### Seed Collection 

*Zanthoxylum rhetsa* (Roxb.) DC is a small deciduous plant that belongs to the family Rutaceae. The fruits of the plant were collected from the central part of Bangladesh in the month of July. The major bioactive constituents of volatile oil obtained from *Zanthoxylum rhetsa* dried fruit are Terpinen-4-ol, α-limonene, β-phellandrene and (+)-sabinene [58]. 

##### Extraction and Isolation

Fresh fruits (500 gm) were cleaned with fresh water and dried in an oven with a constant temperature of 45 °C for 24 h for complete removal of any moisture. Then the seeds were separated from the fruits and put into steam distillation for 6 h. The fine pure oil was separated from sediment crude oil and stored in a screw capped amber glass bottle for further use. The highest yield of volatile oil was obtained from seeds of the dried fruits of *Zanthoxylum rhesta* plants [59].

### 4.2. Chemicals and Reagents

Curcumin (CUR, purity = 99.5%) was supplied by Enzo life Sciences, Lausen, Switzerland. Piperine (PP, purity = 99.8%) and thymoquinone (THQ, purity > 99.8%,) was obtained from Sigma Aldrich, MO, USA. Imwitor 988 (I988, medium chain mono and diglycerides) was obtained from Sasol, GmbH (Werk Witten, Witten, Germany). Apricot kernel oil (APO), avocado oil (AVO) and HCO- 40 (PEG-40-hydrogenated castor oil, HLB = 12.5) were donated by Nikko Chemicals Co. (Tokyo, Japan). Cremophor RH40 (Polyoxyl 40 Hydrogenated Castor Oil-HLB = 14–16, CrRH40) were supplied by BASF, Germany. Transcutol P (TcP) was generously supplied by Gattefossé, France. Simulated intestinal fluid (SIF) powder was purchased from biorelevant.com (London, UK). 

The adsorbents; Aeroperl^®^ 300 (A300) and Neusilin^®^ US2 (NUS) were obtained from Evonik industries (Germany) and Fuji Chemical Industry (Japan), respectively. More detailed information, about the chemical nature and particle properties of the adsorbents, are described in Table 5 [54,55,60].

### 4.3. UHPLC Analysis for CUR and PP Quantification

Chromatographic separation was developed and optimized with respect to the compositions of the stationary and mobile phases, flow-rate, column temperature, and detection wavelength. The study employed the UHPLC system (Thermo scientific, Bedford, MA, USA) consisting of a binary solvent manager equipped with an automatic sample manager (Dionex^®^) and a photodiode array (PDA) eλ detector. The mobile phase involved an isocratic mixture of acetonitrile and ammonium formate (pH 2.5) at 40%/60% *v/v*. The regularly prepared mobile phase was filtered through a 0.20 µm online filter and degassed continuously by the online degasser within the UHPLC system. The flow rate of the mobile phase was 0.3 mL/min. A kinetex^®^, Phenomenex UPLC C18 column (2.1 × 50 mm, 1.6 µm); maintained at 45 °C was used for the analysis. The injection volume was 5 µL and the total run time was 4 min. The detector was set at 428nm and 338nm wavelengths for CUR and PP, respectively. 

### 4.4. Preparation of the CUR–PP-SNEDDS Formulation

Initially, the surfactant, cosurfactant and/or co-solvent were added to the bioactive oil at various ratios. The produced mixture was efficiently homogenized and stored in an airtight 3 mL glass vial until use. To isolate the effect of oil type on formulation performance, each bioactive oil was formulated with a fixed amount of cosurfactant (I988) and surfactant (HCO-40) (F1–F4). In addition, the formulations were optimized by varying surfactants and/or cosolvents (Table 6). The prepared SNEDDS were thoroughly investigated using model drugs CUR and PP. 

### 4.5. Characterization of Liquid CUR–PP SNEDDS Formulation

#### 4.5.1. Equilibrium Solubility of CUR and PP in SNEDDS Formulations

The solubility of CUR and PP within the SNEDDS were investigated using a shake flask method [61]. Excess drug amount was introduced to each sample, which was then thoroughly mixed with a vortex mixer. The samples were effectively incubated at 37 °C for 7 days. Later, the samples were centrifuged in 1.5 mL microfuge tubes at 9800× *g* to separate excess undissolved drug. An aliquot of the supernatant was weighed and diluted with an appropriate solvent. The dissolved CUR and PP were simultaneously analyzed by the developed UHPLC method mentioned above.

#### 4.5.2. Appearance and Morphology 

The anhydrous SNEDDS samples were visually evaluated for homogeneity and appearance. As a preliminary screening, drug-free SNEDDS formulations were mixed with deionized water at 1:100 *w*/*w* ratio to provide fast evaluation of formulation appearance, homogeneity and spontaneity upon aqueous dilution [62,63,64]. Furthermore, SNEDDS formulations were loaded with CUR and PP at ≈80% of their equilibrium solubility and were then subjected to 1:1000 *w*/*w* aqueous dilution prior to formulation evaluation. The dilution factor was selected based on the following facts: (a) in the current study, the dissolution volume of the simulated gastric fluids was 500 mL. (b) The hypothesized amount of drug-loaded SNEDDS, to provide clinically relevant dose of CUR and PP, ranged from ≈250–500 mg. Accordingly, a 1000 fold dilution factor was adopted to mimic the in-vitro dissolution conditions that are hypothesized to mimic the in-vivo dilution conditions upon oral administration [46,65,66]. 

#### 4.5.3. Droplet Size and Zeta Potential

Drug free and CUR–PP loaded SNEDDS samples were diluted 1000-fold with deionized water and mixed prior to measurement. The mean droplet size and zeta potential of the resulted emulsions were measured using a Zetasizer Nano ZS analyzer (Model ZEN3600, Malvern Instruments Co., Worcestershire, UK) [40].

#### 4.5.4. Dynamic Dispersion Studies

Dynamic dispersion studies were performed as a preliminary assessment to evaluate the tendency of drug precipitation during aqueous dispersion, precipitation extent and rate, if any. For the dispersion studies, each of the two drugs CUR and PP were loaded in each formulation at 50–80% of its equilibrium solubility. Next, 250 mg of the drug-loaded formulation was dispersed in 10 mL of fasted and fed state simulated intestinal fluids (FaSSIF and FeSSIF, respectively). The resulted dispersion was subsequently agitated and incubated at 37 °C in a thermostatically controlled water bath for 24 h [67]. Samples were collected at serial time points up to 24 hrs, then centrifuged for 10 min at 9800× *g*. In fact, the supernatant was diluted with appropriate solvent immediately after centrifugation process to inhibit further drug precipitation kinetically. Later, samples were analyzed by UHPLC and the remaining CUR and PP (in solution) were calculated based on the initially analyzed drug concentration in solution [61].

### 4.6. Solidification of CUR-PP Loaded Liquid SNEDDS

Optimal liquid SNEDDS were solidified using the adsorbents Aeroperl 300 and Neusilin US2 (Table 6). The prepared liquid SNEDDS of CUR/PP was added dropwise on the adsorbent in glass mortar (at 1:1 *w*/*w* ratio). The mixture was efficiently mixed until obtaining a uniform solid powder. Subsequently, the solidified SNEDDS were characterized to achieve the optimum formulation, as discussed below.

### 4.7. Characterization of Solid CUR-PP Loaded SNEDDS

#### 4.7.1. Scanning Electron Microscopy (SEM)

Micrographs of the samples were taken using a scanning electron microscope (SEM) (Zeiss EVO LS10; Cambridge, United Kingdom). Samples were fixed on stubs using double sided adhesive carbon tape (SPI Supplies, West Chester, PA, USA) then coated with gold in a Q150R sputter coater unit under vacuum (Quorum Technologies Ltd, East Sussex, United Kingdom) in an argon atmosphere (20 mA) for 60 seconds [68,69].

#### 4.7.2. Differential Scanning Calorimetry (DSC) 

Solidified SNEDDS samples were examined using a differential scanning calorimeter equipped with auto sampler and chiller (DSC8000, Elmer, Waltham, MA, USA). Accurately weighed samples (3–5 mg) were placed in aluminium pans and hermetically sealed using a crimp sealer. The sealed sample pans were heated against a blank aluminium pan from 40 to 250 °C, at a 10 °C/min heating rate and under 50 mL/min nitrogen gas flow rate. The system was calibrated with zinc and indium and data from the thermal analysis were recorded using the Pyris software [13].

#### 4.7.3. X-Ray Powder Diffraction (XRPD)

XRPD samples were evaluated by an Ultima IV diffractometer (Rigaku Corporation, Tokyo, Japan) over 3°−60° 2θ range at 0.5 deg./min scan speed. The tube anode was Cu with Ka = 0.154 nm monochromatized with a graphite crystal. The pattern was collected at tube voltage (40 kV) and tube current (40 mA) in step scan mode (step size 0.02°, counting time 1 second per step) [70,71].

#### 4.7.4. Fourier Transform Infrared Spectroscopy (FTIR)

FTIR studies were performed to examine whether any possible interaction is existing among the drugs CUR, PP and formulations. The chemical properties and complexation of powdered samples was performed by Fourier transform infrared spectroscopy (FTIR Spectrum BX from Perkin Elmer LLC, MA, USA). Pure CUR, pure PP and CUR-PP solid SNEDDS powders were compressed for 5 min at 5 bars on a KBr press and the spectra were scanned on the wavenumber range of 400–4000 cm^−1^.

### 4.8. In-Vitro Dissolution Tests

The dissolution tests were performed using an automated USP Type II dissolution apparatus (UDT-814, LOGAN Inst. Corp., NJ, USA) with a paddle stirrer rotating at 50 rpm speed. The dissolution medium comprised 500 mL of simulated gastric fluid (SGF, pH 1.2, 0.1 N HCl with no enzymes) equilibrated at 37 °C. Weighted amounts of pure drugs and solid SNEDDS (containing 5 mg CUR and 5 mg PP) were filled in “size 00” fish gelatin capsules and placed at the bottom of the vessels using suitable sinker. Samples of 3 mL were withdrawn using a system controller (UDT-800, LOGAN Inst. Corp., NJ, USA) and auto-sampler (UDT-DL, LOGAN Inst. Corp., NJ, USA) at predetermined time intervals 5, 10, 15, 20,30, 60, and 120 min through 10 micron filter tips (LOGAN Instruments Corp., NJ, USA). Freshly prepared dissolution medium was replaced immediately by a system controller and auto-sampler. Samples were centrifuged for 5 min at 9800× *g* then an aliquot of the supernatant was analyzed using the adopted UHPLC^®^ method. After 2 hours, the pH was shifted by adding 250 mL of 0.3 M dibasic sodium phosphate to the dissolution medium to simulate the intestinal pH (SIF, pH 6.8, with no enzymes). The samples were collected at the same time intervals and analyzed as mentioned earlier. The dissolution efficiency (DE)% was utilized to evaluate the dissolution profile of different formulations [72].

### 4.9. Statistical Analysis

QI Macros 2019 software was utilized to analyze the data. One-way analysis of variance (ANOVA) followed by post hoc tests (LSD) were used to compare the droplet size and dissolution results. Two-way ANOVA was used to evaluate influence of adsorbent and pH on dissolution and the influence of drug loading on droplet size and zeta potential. A value for *p* < 0.05 was considered as significant [61].

## 5. Conclusions

The combined dosage form of CUR-PP solid SNEDDS formulation was successfully developed with increased drug solubilization and enhanced dissolution rate. Interestingly, SNEDDS containing BSO presented high CUR-PP solubility, nano-sized self-emulsification along with superior CUR/PP dissolution at both SGF and SIF. NUS solidified SNEDDS showed superior drug dissolution compared to A300 counterparts. Accordingly, the SNEDDS formulation (BSO/I988/TcP/Cr-RH40/NUS, [10/10/5/25/50]) offers a potential oral delivery system for CUR and PP along with the bioactive BSO.

## Figures and Tables

**Table 1:** The most significant biological and pharmacological activities of bioactive materials used in the study.

Ingredient	Biological Activity	References
CUR	✓ Antitumor activities ✓ Antioxidant activity ✓ Anti-inflammatory activity ✓ Memory improvement and anti-depressive ✓ Hepato-protective activity	[20,21,22,23,24,25,26,27,28,29]
PP	✓ Antitumor activity ✓ Antioxidant activity ✓ Anti-inflammatory activity ✓ Antimycobacterial activity ✓ Insecticidal activity	[30]
BSO	✓ Anti-cancer activity ✓ Anti-infective, anthelmintic, antimicrobial, anti-viral, anti-bacterial, antifungal, anti-parasitic and anti-spasmodic activity ✓ Cardiovascular, gastro-protective, hepato-protective, nephron-protective, and testicular-protective activity ✓ Pulmonary-protective activity, broncho-dilating and anti-asthmatic effects ✓ Antioxidant, immune-stimulant, immunomodulatory activity ✓ Hypoglycemic and anti-hypertensive activity ✓ Anti-inflammatory, antihistaminic and analgesic, antipyretic, and anti-ulcerative activity ✓ Neuro-pharmacological, anticonvulsant and antidepressant activity	[31,32,33,34]
APO	✓ Antioxidant activity	[35]
AVO	✓ Antioxidant activity ✓ Promotes the accumulation of HDL cholesterol ✓ Anti-inflammatory activity ✓ Useful in cancer prevention	[36]
ZRO	✓ Anti-inflammatory activity ✓ Antibacterial activity ✓ Antiseptic properties ✓ Antioxidant activity ✓ Antidiarrheal activity	[37,38,39]

**Table 2:** Solubility of CUR and PP in various lipid-based formulations.

No	LFCS Type	Formulation (*w*/*w*)	Solubility (mg/g)	
			CUR	PP
F1	IIIA	APO:I988(7:3)/HCO40 [1:1]	30.4	36.6
F2	IIIA	AVO:I988(7:3)/HCO40 [1:1]	25.2	37.0
F3	IIIA	BSO:I988(7:3)/HCO40 [1:1]	28.2	39.1
F4	IIIA	ZRO:I988(7:3)/HCO40 [1:1]	32.5	44.5
F5	II	ZRO:I988(7:3)/T85 [1:1]	19.0	48.2
F6	IIIB	BSO:I988:TcP(2:2:1)/CrRH40 [1:1]	38.4	45.0

CUR: curcumin, PP: piperine, BSO: black seed oil, I988: Imwitor 988, TcP: Transcutol P, CrRH40: Cremophor RH40, APO: apricot oil, AVO: avocado oil, ZRO: Zanthoxylum rhetsa seed oil and T85: Tween 85.

**Table 3:** Evaluation of drug-free SEDDS/SNEDDS formulations in terms of homogeneity, spontaneity and appearance upon aqueous dispersion.

No	LFCS Type	Formulation (*w*/*w*)	Homogeneity	Spontaneity	Appearance
**F1**	IIIA	APO:I988(7:3)/HCO40 [1:1]	Yes	<1 min	Hazy
**F2**	IIIA	AVO:I988(7:3)/HCO40 [1:1]	Yes	~5 sec	Turbid
**F3**	IIIA	BSO:I988(7:3)/HCO40 [1:1]	Yes	~5 sec	Transparent
**F4**	IIIA	ZRO:I988(7:3)/HCO40 [1:1]	Yes	<1 min	Hazy
**F5**	II	ZRO:I988(7:3)/T85 [1:1]	Yes	<1 min	Turbid
**F6**	IIIB	BSO:I988:TcP(2:2:1)/CrRH40 [1:1]	Yes	~5 sec	Transparent

CUR: curcumin, PP: piperine, BSO: black seed oil, I988: Imwitor 988, TcP: Transcutol P, CrRH40: Cremophor RH40, APO: apricot oil, AVO: avocado oil, ZRO: Zanthoxylum rhetsa seed oil and T85: Tween 85.

**Table 4:** Dissolution efficiency of the representative formulations comprising CUR and PP.

Drug	Formulation	Dissolution Efficiency (%) *
**Curcumin**	Pure Drug Powder	0 *^a^*
	F1A	7.0 ± 0.4 *^b^*
	F1N	35.1 ± 0.6 *^d^*
	F2A	4.1 ± 0.5 *^e^*
	F2N	29.4 ± 0.5 *^f^*
	F6A	7.6 ± 1.6 ^*b*^
	F6N	45.7 ± 0.6 *^c^*
**Piperine**	Pure Drug Power	13.9 ± 1.7 *^i^*
	F1A	36.4 ± 3.8 *^iii^*
	F1N	42.7 ± 1.8 *^iv^*
	F2A	42.6 ± 3.8 *^iv^*
	F2N	51.5 ± 4.3 *^v^*
	F6A	67.8 ± 3.8 *^ii^*
	F6N	65.7 ± 1.0 *^ii^*

* Data are expressed as mean ± SD. Different superscripted letters indicate statistical significance between formulations (*p* < 0.05).

**Table 5:** The chemical nature and particle properties of Aeroperl 300 and Neusilin US2.

Adsorbent	Aeropearl^®^ 300	Neusilin^®^ US2
Chemical Composition [60]	Granulated fumed silica	Magnesium aluminometasilicate
Chemical Formula	SiO_2_	Al_2_O_3_·MgO·1.7SiO_2_·xH_2_O
Specific Surface Area (m2/g) [54]	300	300
Particle Size (µm) [60]	30–40	60–120
Pore Volume (ml/g) * [54]	2.2	4
Peak Pore Size (nm) ** [54]	≈25	≈800
Predominant Type of Pores [54,55]	Mesoporous (2–50nm)	Macroporous (>50nm)

* based on the mercury intrusion-extrusion technique. ** based on the highest intensity pore size within the size distribution curve generated by the mercury intrusion-extrusion technique

**Table 6:** The compositions of lipid-based formulations.

Formulation	LFCS Type				Excipients Percentage (*w*/*w* %)							Total	
		APO	AVO	BSO	ZRO	I988	TcP	HCO-40	Cr-RH40	T85	NUS	A300	
F1	IIIA	35	-	-	-	15	-	50	-	-	-	-	100
F2	IIIA	-	35	-	-	15	-	50	-	-	-	-	100
F3	IIIA	-	-	35	-	15	-	50	-	-	-	-	100
F4	IIIA	-	-	-	35	15	-	50	-	-	-	-	100
F5	II	-	-	-	35	15	-	-	-	50	-	-	100
F6	IIIB	-	-	20	-	20	10	-	50	-	-	-	100
F1A	IIIA	17.5	-	-	-	7.5	-	25	-	-	-	50	100
F2A	IIIA	-	17.5	-	-	7.5	-	25	-	-	-	50	100
F6A	IIIB	-	-	10	-	10	5	-	25	-	-	50	100
F1N	IIIA	17.5	-	-	-	7.5	-	25	-	-	50	-	100
F2N	IIIA	-	17.5	-	-	7.5	-	25	-	-	50	-	100
F6N	IIIB	-	-	10	-	10	5	-	25	-	50	-	100

**Abbreviations:** LFCS, lipid formulation classification system; BSO: black seed oil, I988: Imwitor 988, TcP: Transcutol P, CrRH40: Cremophor RH40, APO: apricot oil, AVO: avocado oil*,* ZRO: essential oil of *Zanthoxylum rhetsa* seeds obtained by steam distillation and T85: Tween 85, NUS Neusilin US2, A300: Aeroperl 300.
